# A sustained-release phospholipid-based phase separation gel loaded with berberine for treating rheumatoid arthritis

**DOI:** 10.3389/fphar.2023.1210129

**Published:** 2023-07-20

**Authors:** Xiong Peng, Yuping Yang, Chenqi Guo, Qin He, Yan Li, Tao Gong, Jia Li

**Affiliations:** ^1^ Key Laboratory of Drug-Targeting and Drug Delivery System of the Education Ministry and Sichuan Province, Sichuan Engineering Laboratory for Plant-Sourced Drug and Sichuan Research Center for Drug Precision Industrial Technology, West China School of Pharmacy, Sichuan University, Chengdu, China; ^2^ Sichuan Institute for Drug Control, NMPA Key Laboratory for Quality Control and Evaluation of Vaccines and Biological Products, Chengdu, China; ^3^ National Clinical Research Center for Oral Diseases, West China Hospital of Stomatology, Sichuan University, Chengdu, China

**Keywords:** rheumatoid arthritis, berberine, phospholipid-based phase separation gel, sustained release, drug delivery

## Abstract

Berberine (BBR) has a long history of use in the treatment of Rheumatoid arthritis (RA) and is considered one of the most promising natural product for the treatment of RA. However, oral administration of berberine has low bioavailability and requires frequent administration, resulting in poor patient compliance. In this study, we developed a BBR-loaded phospholipid-based phase separation gel (BBR-PPSG) to achieve sustained drug release and long-term therapeutic effect. The stability of BBR-PPSG was verified and it was found that it can be stored for a long time. The pharmacokinetic study on rats and rabbits showed that BBR-PPSG not only achieved 1-month of sustained release, but also significantly increased the area under the curve (AUC) by nearly 9-fold and prolonged the half-life (t_1/2_) by 10-fold. By constructing rat and rabbit models of RA, we also proved that BBR-PPSG administration once a month effectively alleviated joint swelling, and significantly reduce TNF-α levels in AIA rats and OIA rabbits. Histopathological analysis of rabbit joint sections revealed that after intra-articular injection of BBR-PPSG, the synovial cell layer remained intact, while in the model group, the synovial cells were significantly reduced and exhibited necrosis. MicroCT data analysis showed that the values of Tb.N and Tb. Sp in the BBR-PPSG group were significantly better than those in the model group (*p* < 0.05). This study addressed the limitations of frequent administration of BBR by developing a phospholipid-based phase separation gel system for berberine delivery, achieving long-term sustained release. The BBR-PPSG demonstrated good biocompatibility, simple preparation and excellent stability, thus holding potential as a novel pharmaceutical formulation for RA treatment.

## 1 Introduction

Rheumatoid arthritis (RA) is a systemic immune disease characterized by chronic inflammation of joint tissues, and its primary target is synovial tissues and articular cartilage. It can affect people of all races, regions, and ages worldwide, with a global overall prevalence of approximately 1%, being more common in the age range of 40–50 years (van der Horst-Bruinsma et al., 2009; Smolen et al., 2016; Wang K. et al., 2017). The main clinical symptoms were arthrocele, dysarthrose, pain and poor flexion and extension. As the disease progresses, it can lead to synovitis with cartilage and bone destruction. In severe cases, patients can suffer joint functional disability, organ failure and even death. It not only seriously affects the quality of life, but also causes some patients to lose their ability to work for 10 years, becoming the biggest killer of physical disability (Gwinnutt et al., 2022). Unfortunately, the pathogenesis of rheumatoid arthritis is still unclear. Presumably, it may be related to such factors as immunity, gene, environment, and infection (Yu et al., 2013). Rheumatoid factor (RF) is one of the important serum criteria for the diagnosis of rheumatoid arthritis, and its positive rate in rheumatoid arthritis is about 80% (Wu et al., 2021). In RA inflammatory state, various pathophysiological changes of RA are mediated by secretion of various pro-inflammatory cytokines (TNF-α, IL-1β, IL-17 and IL-23) and chemokines (RANKL, GM-CSF and MMPs). TNF-α and IL-1β are the major cytokines in RA synovium (Rockel and Kapoor, 2016; Asif et al., 2017). In addition, erythrocyte sedimentation rate increased significantly during the active phase of rheumatoid arthritis (Wasserman, 2011). RA cannot be cured completely so far. Clinical treatment mainly aims to relieve inflammation and control the development of the disease. Long-term oral or intra-articular injection is usually used.

Berberine (BBR), an isoquinoline alkaloid extracted from Ranunculaceae plants such as Coptis chinensis, is widely sourced, easily extracted and broadly used in clinical treatment (Huang D. N. et al., 2021). Studies have showed that berberine has anti-inflammatory, antibacterial, hypoglycemia, antiarrhythmic and immunomodulatory effects, and with further research, more pharmacological effects have been discovered (Qiu et al., 2014; Zhang T. et al., 2015; Pozsgay et al., 2017). Berberine has been used in clinical treatment of RA for many years and is considered one of the most promising natural product derivative drugs for treating RA (Huang S. M. et al., 2021). In clinical practice, berberine is often used in combination with other anti-RA drugs to enhance efficacy and reduce adverse reactions. Clinical trials have shown that combined treatment with berberine and other anti-RA drugs increases the effective rate by about 15% and reduces adverse reactions by 7% compared with the non-berberine group (Wang et al., 2013). In recent years, many studies have been conducted to explore the mechanism of berberine’s anti-RA effects. Berberine exerts therapeutic effects from multiple pathways and targets, including regulating immune response, inhibiting synovial hyperplasia, inhibiting angiogenesis, reducing bone erosion, and regulating gut microbiota (Huang D. N. et al., 2021).

Although increasing evidence suggests that BBR can combat RA both *in vivo* and *in vitro*, there are still some drawbacks to its clinical use. Berberine is usually formulated as regular tablets or capsules, which need to be taken three times a day for a long time (Liu et al., 2016). Frequent administration not only constrains patient compliance, but also presents limitations such as low bioavailability and gastrointestinal discomfort (Liu et al., 2010; Wang X. et al., 2017). Sustained-release formulations with fewer doses, good compliance, local administration, and reliable quality and safety are expected to become a long-term medication option for RA patients.

The phospholipid-based phase separation gel (PPSG) is a sustained-release drug delivery system developed by our research group. PPSG consists of ethanol, medium chain triglyceride and 70% (w/w) phospholipid (Zhang et al., 2019a; Zhang et al., 2020; Wang et al., 2016). *In vitro*, PPSG is a flowing liquid and the viscosity is affected by the proportion of prescription, which is about 165 to 500 cP (Zhang et al., 2019b; Han et al., 2016). After injection into the body, PPSG gradually semi-solidifies with the exchange of ethanol and body fluids and transforms into a gel that adheres to the tissue to become a “drug reservoir”, slowly degrades in the body, and releases the drug into the injection area. In addition, PPSG can be degraded completely in about 1 month, avoiding the need for a second invasive surgery. PPSG is absorbable, and all raw materials are commonly used non-toxic reagents, so its side effects are also minimal (Zhang et al., 2019b).

Combined with previous studies, we intend to prepare berberine phospholipid-based phase separation gel (BBR-PPSG). It is expected that inject BBR-PPSG into the articular cavity can reduce the frequency of administration and systemic toxicity, improve bioavailability. Furthermore, the pharmacokinetic parameters and the comprehensive therapeutic effects of BBR-PPSG in different animal models of RA will be verified.

## 2 Materials and methods

### 2.1 Materials

Berberine Chloride Hydrate was purchased from Shanghai Aladdin Bio-Chem Technology Co., LTD. (Shanghai, China); Soyabean lecithin S100 was purchased from Lipoid Co. LTD. (Ludwigshafen, Germany); Injection-grade medium-chain triglyceride (MCT) was obtained from Beiya Medical Oil Co. Ltd. (Tieling, China); HPLC-grade Ethanol was purchased from Tianjin Kemiou Chemical Reagent Co., LTD. (Tianjin, China).

Enzyme-linked immunosorbent assay (ELISA) kits for detecting rat TNF-α and IL-1β levels were purchased from Ruixin Biotech Chengdu, China. ELISA kits to determine rabbits’ TNF-α levels and RF were purchased from Signalway Antibody (MD, United States). Complete Freund’s adjuvant (CFA, 1 mg/ml of heat-killed mycobacteria) was purchased from Sigma-Aldrich Chemical Co (Shang Hai, China). CFA (10 mg/ml) was purchased from Chondrex (Washington DC, United States). Ovalbumin (OVA) was procured from Sigma-Aldrich Chemical Co.

### 2.2 Experimental Animals

Male Sprague-Dawley rats (160–180 g) were raised in specific pathogen-free condition with a relative humidity of 55% (45%–70%), a 12-h light/dark cycle, and a standard temperature-controlled environment (22°C ± 2°C). New Zealand rabbits (4-week-old, 1.8–2.0 kg) were raised in specific pathogen-free condition with a relative humidity of 65% (55%–70%), a 12-h light/dark cycle, and a standard temperature-controlled environment (20°C ± 5°C). All rabbits were allowed free access to standard food and water. All animals were obtained from Chengdu DaShuo Experimental Animal Co., Ltd. (Chengdu, China).

All animal protocols and experiments were carried out in accordance with the requirements of the National Act on the Use of Experimental Animals (China) and were approved by the Animal Ethics Committee of Sichuan University (SYXK-Chuan-2018–113).

### 2.3 Preparation of PPSG

Blank PPSG was prepared by simple magnetic stirring with S100, MCT and 85% ethanol at the ratio of 70:15:15 for 2 h. Then, berberine powder was added into the gel system, sealed, ultrasonic at 50°C for 10 min, and stirred for 5 min to form berberine phospholipid-based phase separation gel.

Preparation of berberine injection: Berberine hydrochloride was dissolved by DMSO, and then diluted with water to obtain berberine injection (BBR-I).

### 2.4 Stability of BBR-PPSG

The prepared BBR-PPSG was filled with nitrogen, sealed with gland and stored for 1 year at 4°C and 37°C respectively. BBR content was measured at the 1st, 3rd, 5th, 10th, 15th, 20th, 25th, 30th, 60th, 90th, 120th, 240th, and 360th days. The content of BBR was determined by HPLC on KromasilC18 column (4.6 × 250 mm, 5 µm) using 50 mL acetonitrile and 0.02 mol/L potassium dihydrogen phosphate solution (25:75) as mobile phase. The flow rate was 1.0 mL/min, column temperature was 25°C and detection wavelength was 424 nm.

### 2.5 Pharmacokinetics of BBR-PPSG

Healthy male SD rats were randomly divided into three groups (n = 6): BBR-I-O group (oral berberine injection), BBR-I-S group (subcutaneous injection of berberine injection), BBR-PPSG-S group (subcutaneous injection of berberine phospholipid-based phase separation gel). The concentration of BBR in both BBR-I and BBR-PPSG was 1 mg/ml, and each group was given a single dose of 1 ml. Each group was administered only once. 500μL of blood was collected by blood collection vessel at 0.5, 1, 2, 4, 8, 12 h, 1, 2, 3, 4, 8, 10, 15, 20, 25, 27, and 30 days after medication, then centrifuged immediately at 6000 rpm for 5 min. Plasma samples (100 μL) were collected and stored at −40°C. The plasma BBR concentration was determined by liquid chromatography mass spectrometry (LC-MS). The conditions for liquid chromatography were as follows: C18 column; methanol-water (containing 0.1% formic acid) (v/v) was mobile phase with gradient elution; the flow rate was 0.2 mL/min; the sample size was 10 μ; the column temperature was 25°C. The mass spectrum conditions were as follows: electrospray ion source (ESI); ion polarity; positive ion mode; multi-response monitoring (MRM); Berberine [M + H] + m/z 337.2→291.1 was the target ion pair.

Healthy rabbits were randomly divided into two groups (n = 6): BBR-I-A group (intra-articular injection of BBR injection), BBR-PPSG-A (intra-articular injection of berberine phospholipid-based phase separation gel). The concentrations of BBR in BBR-I and BBR-PPSG were both 1 mg/ml. Each knee joint of the hind legs was administered once, 0.5 ml/joint. Plasma BBR concentration was measured at 0.25, 0.5, 1, 2, 4, 8, 12 h, 1, 2, 3, 5, 8, 10, 15, 21, and 25 days after medication. The collection and detection methods were consistent with those of rats.

### 2.6 Therapeutic effects of BBR-PPSG in rats

#### 2.6.1 AIA rat model

As one of the most common animal models, adjuvant induced arthritis (AIA) rat models were widely used in the evaluation of anti-rheumatoid drugs and rheumatoid arthritis. Its clinical and histological features were similar to those of human rheumatoid arthritis (Whitehouse, 2007). The AIA rat model was established according to Gong’s research (Gong et al., 2020). The model was induced by intradermal injection of CFA containing 10 mg/mL heat-killed mycobacteria into the two hind paws of rats respectively.

#### 2.6.2 Groups

The AIA rats were randomly divided into 4 groups (n = 7): model group (subcutaneously injected normal saline, 1 ml/d, for 30 days), BBR-I-O group (oral BBR injection, 180 mg kg^−1^·d^−1^, 1 ml/d for 30 days), BBR-I-S group (subcutaneous injection of BBR injection, 4.896 mg kg^−1^·d^−1^, 1 ml/d for 30 days), and BBR-PPSG-S group (subcutaneous injection of BBR-PPSG solution, 146.88 mg/rat, 1 ml, only once). The BBR-PPSG-S group was injected only once on the back. 7 healthy rats were selected as controls. All 5 groups of rats were weighed at day 1, 4, 7, 10, 13, 16, 20, 25 and 30, and the thickness of their paws was measured with vernier calipers.

#### 2.6.3 TNF-α and IL-1β level in serum

Blood Samples were collected by orbit on day 25. Serum samples were obtained by centrifuging at 5000 rpm for 8 min and stored at −80°C for use. Serum TNF- α and IL-1 β levels were measured by ELISA.

### 2.7 Therapeutic effects of BBR-PPSG in rabbits

#### 2.7.1 OIA rabbit model

According to Dumonde’s research, OVA was added to the rabbit RA model in order to improve RA symptoms in rabbits. The local joint inflammatory response was induced and maintained after multiple injections of OVA to ensure that the rabbit RA model was eligible for long-term sustained-release treatment of PPSG (Dumonde and Glynn, 1962). Oil induced arthritis (OIA) rabbit modeling involved two steps: OVA and CFA mixed emulsion was injected into the back of rabbits once a week for 2 weeks; Then the mixed emulsion was injected into the knee cavity once a week for 2 weeks. Blood samples of rabbits were collected from auricular veins before and at 4 and 6 weeks after modeling to detect rheumatoid factor concentration.

#### 2.7.2 Groups

After the establishment of OIA model, the rabbits were randomly divided into 3 groups (n = 6): model group (posterior knee joint cavities were injected with saline, 0.5 ml/joint, for 28 days), BBR-I-A group (posterior knee joint cavities were injected with BBR-I, 4.59 mg kg^−1^·d^−1^, 0.5 ml/joint, for 28 days), and BBR-PPSG-A group (posterior knee joint cavities were injected with BBR-PPSG, 137.7 mg/joint, 0.5 ml/joint, only once). Seven healthy rabbits were included as control. The thickness of the knee joint was measured with a vernier caliper on the 1st, 3rd, 6th, 9th, 12th, 15th, 21st, and 28th days after administration.

#### 2.7.3 Histopathological examination

Two days after the last administration of BBR injection to the knee joint, all rabbits were killed by air injection through the ear vein, and the muscle tissue around the knee joint and other tissues were taken for histological analysis. The tibia and femur about 3 cm above and below the patella were removed to obtain a complete knee joint cavity. The hind leg knee joints of each rabbit were fixed in 4% paraformaldehyde, decalcified in 15% (w ≤ v) ethylenediaminetetraacetic acid solution for 2 months, embedded in paraffin, and sliced to a thickness of about 5 μm. Finally, hematoxylin-eosin (HE) staining was used to prepare pathological tissues. The histopathological morphology of the knee joint was observed under an optical microscope (Olympus BX53, Tokyo, Japan).

#### 2.7.4 TNF- α level and erythrocyte sedimentation rate

Rabbits’ blood sample were collected from the auricular veins on day 28, centrifuged at 5000rpm for 8min to obtain serum, and then stored at −80°C for use. Serum TNF- α level was measured by ELISA kits, and erythrocyte sedimentation rate was determined by sedimentation tube.

#### 2.7.5 Micro-CT analysis

Micro-CT (VivaCT 80, SCANCO Medical AG, Switzerland) was used to scan the knee joints of each group at 70 kV, 114 μA, 8 W, and 35 μm resolution. The dataset was reconstructed to obtain 3D images of the distal femur joints and trabeculae. Osseous function parameters included bone mineral density (BMD), bone volume to total volume ratio (BV/TV), bone surface area to bone volume ratio (BS/BV), trabecular number (Tb. N), trabecular thickness (Tb. Th), and trabecular separation (Tb. Sp). Three rabbits were randomly selected from each group, and the micro-CT analysis was performed on one hind leg knee joint of each rabbit.

### 2.8 Statistical analysis

Results were presented as mean ± standard deviation (SD). Pharmacokinetic parameters were calculated using nonlinear regression in Drug and Statistics software 2.0 (Mathematical Pharmacology Professional Committee of China, Shanghai, China). All other statistical analyses were performed using GraphPad Prism 6.0 (GraphPad Software, CA, United States). Differences between two groups were assessed using Student’s t-test; differences between multiple groups were assessed using one-way analysis of variance (ANOVA). Difference was considered statistically significant when *p* is less than 0.05.

## 3 Results

### 3.1 Preparation and characterization of BBR-PPSG

**FIGURE 1:**
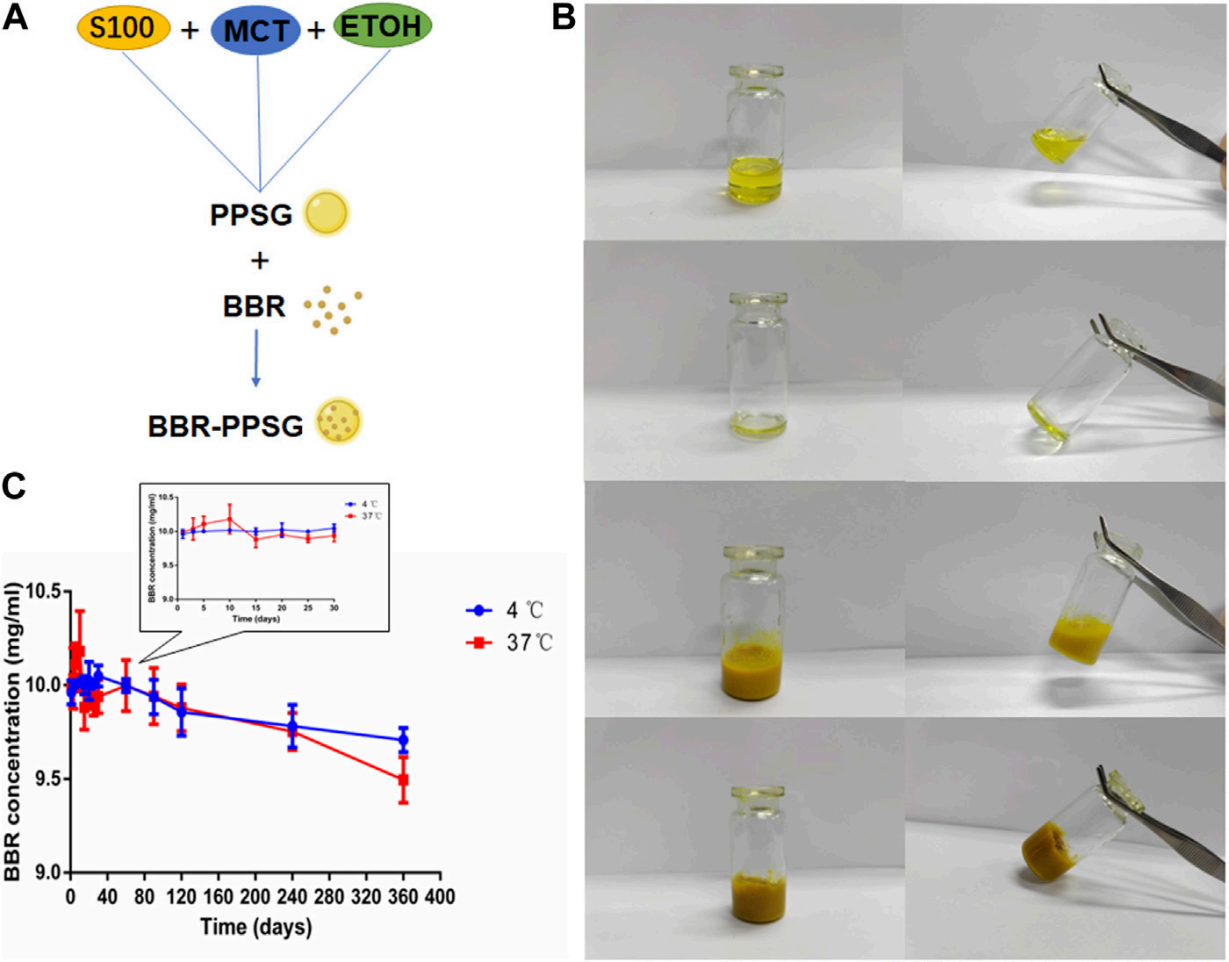
Preparation and characterization of BBR-PPSG. **(A)** Preparation of BBR-PPSG. **(B)** Photographs of PPSG and BBR-PPSG. **(C)** Stability of BBR-PPSG.

The preparation of BBR-PPSG is shown in Figure 1A. Briefly, BBR-PPSG was prepared by magnetic stirring. BBR was uniformly suspended in the gel system. Before the phase transition, the blank PPSG appeared as a transparent yellow solution with good fluidity (Figure 1B). It changed liquidity after losing ethanol. Due to the insolubility of BBR in ethanol, BBR-PPSG appeared as a yellow suspended solution. BBR-PPSG also lost fluidity during the evaporation of ethanol.

The stability experiment of BBR-PPSG (10 mg/ml) stored at 4°C and 37°C was observed. As shown in the Figure 1C, there was little change in concentration in both groups after 3 months, with a decrease in concentration of about 0.6%. One year later, the decrease was 2.9% at 4°C and only 5.0% at 37°C. Moreover, there was no sedimentation or stratification. The results show that BBR loading in phospholipid gel has good stability. The preservation of BBR-PPSG is not affected by temperature, and can be stored at room temperature and low temperature for a long time.

### 3.2 Pharmacokinetics

**FIGURE 2:**
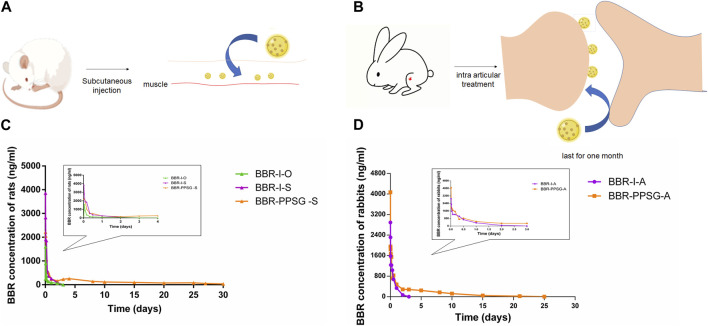
Pharmacokinetic results of BBR-PPSG in rats and rabbits. **(A)** Schematic diagram of subcutaneous injection in rats. **(B)** Schematic diagram of joint injection in rabbits. **(C)** Pharmacokinetic curve in rats (n = 6). **(D)** Pharmacokinetic curve in rabbits (n = 6).

We have studied the release of BBR-PPSG as a long-term sustained release drug in rats and rabbits (Figures 2A, B). Among the three groups of rats, the blood drug concentration in BBR-I-S group was highest 30 min after administration, which subsequently decreased and was undetectable on the second day (Figure 2C). The BBR-I-O group had the lowest concentration among the three groups and failed to detect BBR after 48 h. The BBR-PPSG-S group had higher blood concentration than the other two groups after 24 h. BBR-PPSG was released continuously for 30 days, and the plasma BBR concentration at 20th days was close to that at 10th day and over 40 ng/ml at 30th days. Compared with the other two groups, the AUC and t_1/2_ of BBR-PPSG-S group were significantly higher, while the T_max_ value was reduced (Table 1).

**TABLE 1:** Pharmacokinetic data of rats.

Parameter	BBR-I-O	BBR-I-S	BBR-PPSG-S
AUC _(0-t)_ ng/L*h	7654.13 ± 638.47	23,451.97 ± 7387.07	94,679.83 ± 7280.92
t_1/2_ h	19.4 ± 6.96	14.45 ± 0.52	215.87 ± 138.91
T_max_ h	1.00	1.40 ± 1.48	0.70 ± 0.27
C_max_ ng/L	1607.04 ± 221.91	5001.54 ± 1274.02	2425.81 ± 220.12

The pharmacokinetics results of rabbits were shown in Figure 2D. During the first 2 days, the plasma concentration curves of the two groups were very similar. However, after 2 days, the BBR concentration in BBR-PPSG-A group was significantly higher than that in BBR-I-A group. Moreover, BBR was still detectable in plasma up to 25 days in the BBR-PPSG-A group. Compared with BBR-I-A group, the BBR-PPSG-A group showed a markedly higher AUC and t_1/2_ values (Table 2).

**TABLE 2:** Pharmacokinetic data of rabbits.

Parameter	BBR-I-A	BBR-PPSG-A
AUC _(0-t)_ ng/L*h	24,810.21 ± 1990.16	87,734.04 ± 6819.04
t_1/2_ h	11.15 ± 1.91	98.44 ± 14.71
T_max_ h	0.30 ± 0.11	0.25
C_max_ ng/L	3177.57 ± 943.79	4078.12 ± 1660.84

Poor bioavailability of berberine was seen in rats, consistent with previous studies. Berberine has extremely low water solubility, and oral administration is the main factor limiting its application (Feng et al., 2021). Berberine injection can improve the bioavailability to a certain extent, but the rapid metabolism after injection requires frequent administration. Preparing berberine as a sustained-release gel not only achieved 1-month sustained release but also greatly improved its bioavailability.

### 3.3 Therapeutic efficacy in rats

#### 3.3.1 Model verification

**FIGURE 3:**
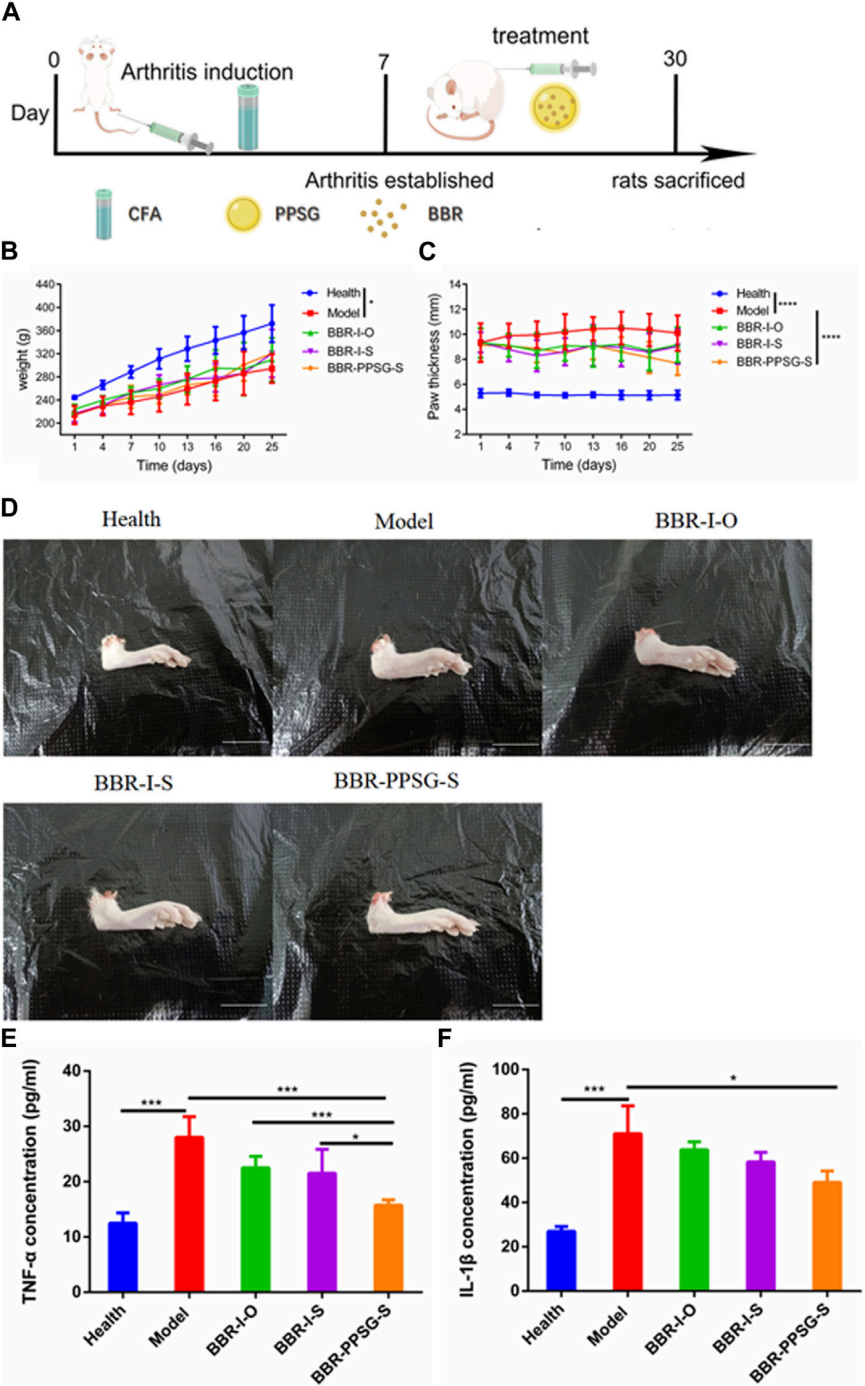
Evaluation of therapeutic effect on AIA rats’ model. **(A)** Schematic diagram of arthritis induction and subcutaneous injection in rats. **(B)** Bodyweights of rats. **(C)** Paw thicknesses of rats. **(D)** Representative pictures of hind paw (Scale bar = 20 mm). **(E)** Serum levels of TNF-α. **(F)** Serum levels of IL-1β. Data represent mean ± SD (n = 7). *p < 0.05, **p < 0.01, ***p < 0.001, ****p < 0.0001.

Figure 3A shows the establishment of AIA rat model. The weight growth of rats in each group after administration was shown in Figure 3B. The weight of AIA rats increased slowly compared with the health group, and there was no significant difference among the AIA groups. The thickness of paw is also more swollen in AIA rats, as shown in Figure 3C. Figure 3D showed the paw photos of rats in each group on the 25th days. AIA rats were more swollen than the healthy rats. On the 25th days, the TNF-α and IL-1β values of rats in AIA groups were significantly higher than those of healthy rats (Table 3). In terms of body weight, paw thickness and cytokine level, there were statistical differences between the model groups and the healthy group, indicating that the AIA rats’ model was established successfully.

**TABLE 3:** TNF-α and IL-1β values of rats in different groups.

	Health	Model	BBR-I-O	BBR-I-S	BBR-PPSG-S
TNF-α (pg/ml)	12.63 ± 2.00	28.22 ± 3.56	22.68 ± 2.00	21.95 ± 3.98	16.08 ± 1.25
IL-1β (pg/ml)	27.34 ± 1.96	71.69 ± 12.43	64.23 ± 3.67	59.01 ± 4.54	49.46 ± 5.15

#### 3.3.2 Efficacy evaluation

In terms of weight gain in rats, the body weight of the three medication groups was higher than that of the model group. There was no significant difference in body weight at day 25 among the three groups. Compared with the model group, the swelling degree of the paw in the three medication groups was lighter, and the comparison of data also had statistical differences, but there was no statistical difference among the three medication groups. In addition, compared with the model group, the cytokine levels in the three medication groups were also improved, and the values of TNF-α and IL-1β were statistically different between the BBR-PPSG-S group and the model group. There were statistical differences in TNF-α level between the BBR-PPSG-S group and the other two groups, while IL-1β values had no statistical difference.

In general, the treatment groups improved the RA status of rats, verifying the therapeutic effect of BBR on RA. Among the three medication groups, BBR-PPSG-S group had the heaviest body weight, the thinnest paw thickness, and the lowest TNF-α and IL-1β values. Except for TNF-α, there was no significant difference among the three groups. These results indicated that compared with daily continuous administration, BBR-PPSG not only achieved the goal of sustained release for nearly 1 month, but also had similar therapeutic effect to daily administration.

### 3.4 Therapeutic efficacy in rabbits

#### 3.4.1 Validation of the OIA rabbit model

**FIGURE 4:**
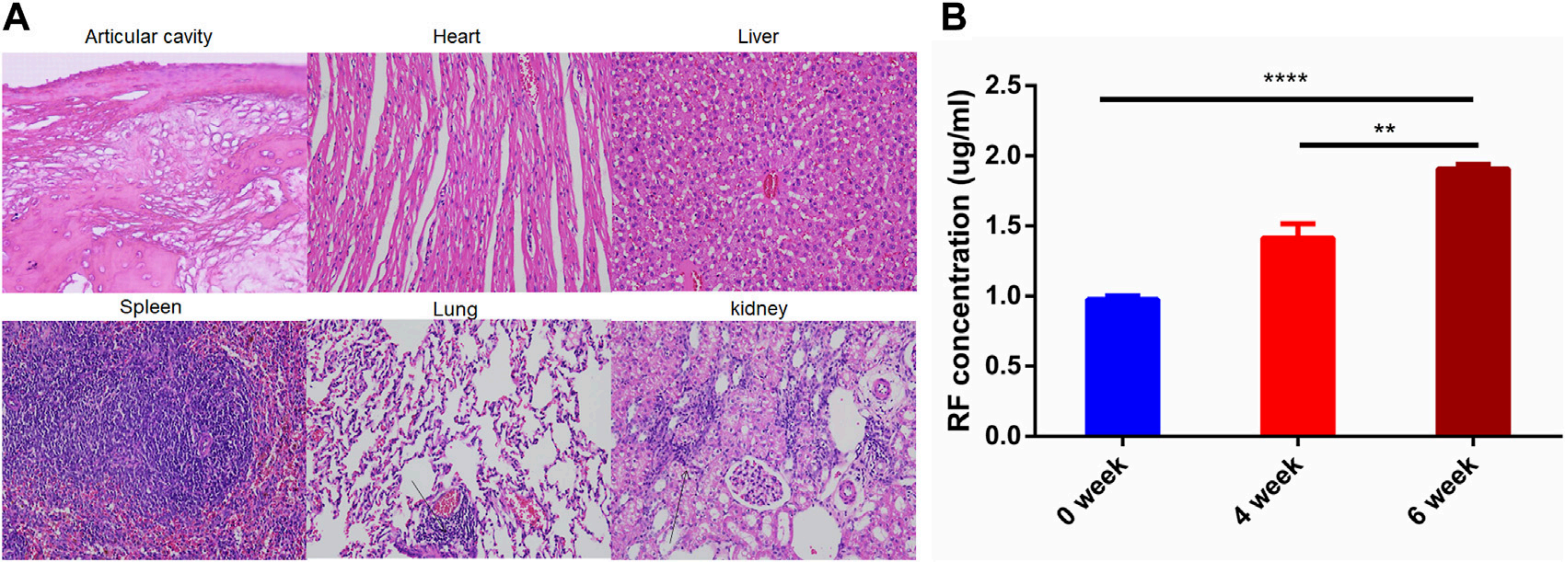
OIA Rabbit Model. **(A)** HE staining images of joint cavity, heart, liver, spleen, and kidney in OIA rabbits **(B)** RF concentration OIA rabbits. ****p < 0.0001.

**FIGURE 5:**
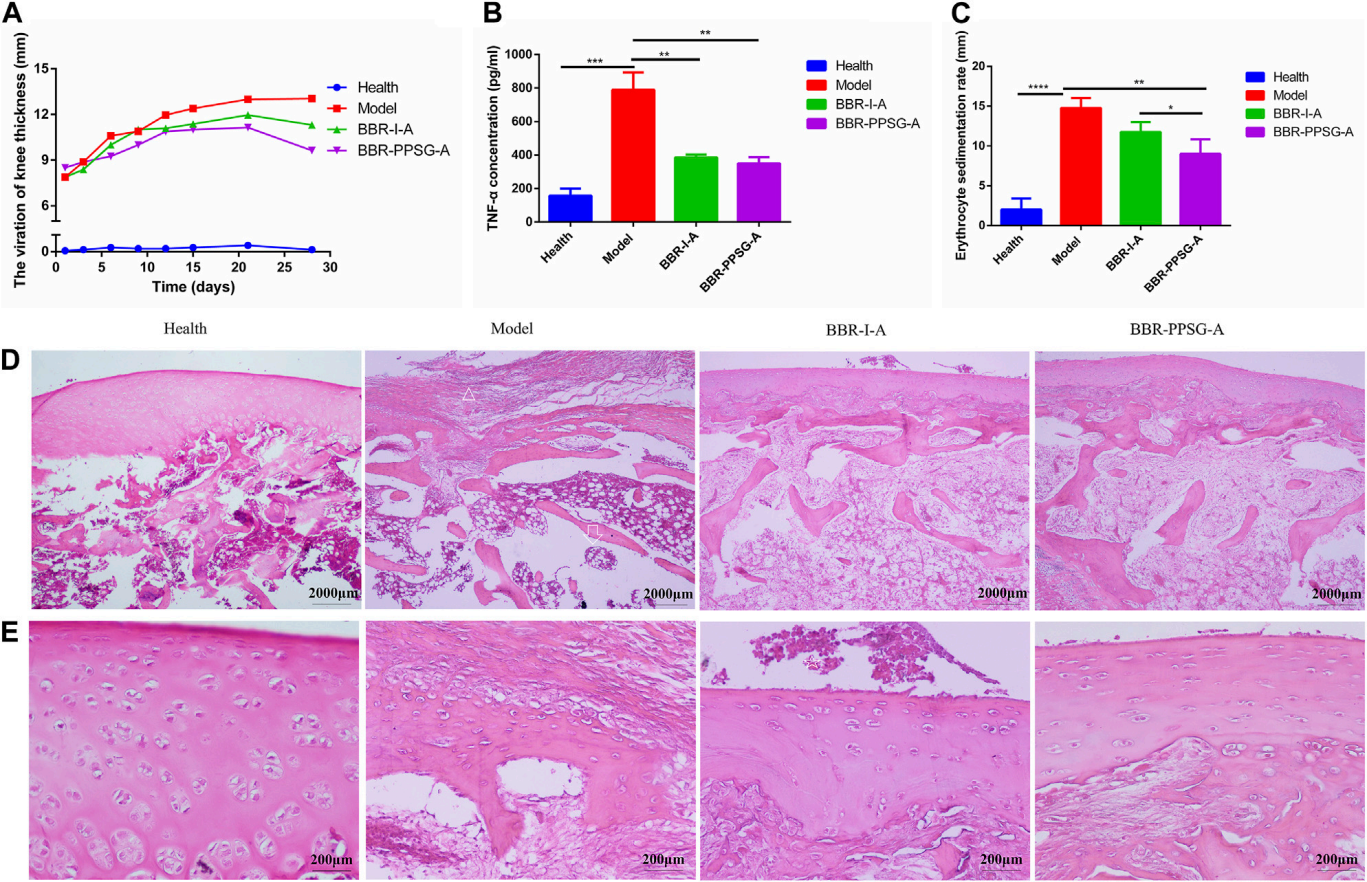
Evaluation of therapeutic effect on OIA rabbits. **(A)** Thickness of rabbit’s knee joint. **(B)** TNF-α level in rabbit serum. **(C)** Erythrocyte sedimentation rate. **(D)** HE-stained pictures of knee joints (Scale bar = 50 µm) **(E)** HE-stained pictures of knee joints (Scale bar = 200 μm). Results are presented as mean ± SD (n = 6).

Figure 4A shows the histopathological results of rabbits in the model group. Joint surface defects, synovial hyperplasia, cellulose exudation, and bone tissue destruction were observed in OIA knee section of rabbit model. There was obvious inflammatory cell infiltration in lung tissue and individual inflammatory cell infiltration in liver tissue. The kidney sections showed flattened, detached, loose, and edematous renal tubular epithelial cells, with evident inflammatory cell infiltration. In addition to the obvious histopathological features, the concentration of rheumatoid factor (RF) also increased with the degree of arthritis (Figure 5B; 0 week: 0.97 ± 0.02 µg/ml, 4 weeks: 1.41 ± 0.10 µg/ml, 6 weeks: 1.91 ± 0.03 µg/ml). The above results indicate that the OIA rabbit model was successfully established.

#### 3.4.2 Inflammation relief effect

The effect of berberine in each group was shown in Figure 5. At the end of treatment, the thickness of the knee joint in three groups were as follows: BBR-PPSG-A group < BBR-I-A group < model group (Figure 5A). Compared with the two treatment groups, the BCR-PPSG-A group had the lowest thickness during the study period, and there was no statistical difference between the two groups.

The increase of TNF-α level in the rheumatoid inflammatory microenvironment leads to the gradual increase of synovial hyperplasia fluid, resulting in synovial hyperplasia and articular cartilage destruction. TNF-α, as one of the criteria for the treatment of RA, is also considered one of the key indicators for the treatment of arthritis in advanced AIA rats and OIA rabbits. The TNF-α level of rabbits in each group was detected, and compared with the model group, the TNF-α level of rabbits in the two groups were significantly lower, with statistical differences. Although the lowest value was found in the BBR-PPSG-A group, there was no statistical difference between the two groups (Figure 5B). In comparison with the erythrocyte sedimentation rate of the model group, the two medication groups were lower, and only BBR-PPSG-A group and the model group had statistical differences. However, there was also a statistical difference between the two treatment groups, indicating that the effect of the BBR-PPSG-A group was superior to that of the BBR-I-A group (Figure 5C). The values of TNF-α and ESR in all treatment groups were lower than those in model group, indicating that inflammation was effectively controlled during treatment.

In order to further prove that BBR-PPSG can control inflammation and reduce cartilage destruction, rabbit knee joint sections were taken for HE staining at the end of the study to observe histopathological changes and inflammatory status. HE stained sections of the model group showed severe synovial hyperplasia, inflammatory synovial cell infiltration with bone and cartilage destruction, complete disappearance of some articular cartilage and bone tissue, and tendon fibrosis (Figures 5D, E). As marked with the triangle, few chondrocytes were found in the cartilage layer, where 6-8 layers of fibrocytes appeared instead, indicating fiber repair hyperplasia after chondrocyte necrosis. Trabecular collapse was pointed with arrows. The images showed that synovial inflammation and cartilage loss were significantly reduced in the treatment group compared to the model group, and the therapeutic effect was better in the BBR-PPSG-A group than in the BBR-I-A group (Figure 5E). Combined with Elisa results, it was not difficult to find that the inflammatory synovial microenvironment was gradually restored after BBR-PPSG-A treatment, and the cartilage injury was effectively reversed, with only mild synovial hyperplasia (Table 4). These results indicate that BBR-PPSG not only achieves the purpose of sustained release, but also has better therapeutic effect than BBR-I, effectively alleviating arthritic synovial inflammation and cartilage destruction in OIA rabbits.

**TABLE 4:** TNF-α and ESR values of rabbits in different groups.

	Health	Model	BBR-I-A	BBR-PPSG-A
TNF-α (pg/ml)	156.37 ± 42.31	789.51 ± 105.07	385.24 ± 17.55	349.47 ± 36.86
ESR (mm)	1.76 ± 1.15	14.76 ± 1.08	11.80 ± 1.22	9.04 ± 1.59

#### 3.4.3 Bone preservation

Osteoporosis is widespread in RA patients because RA severely destroys osteoblasts and enhances bone resorption (Auréal et al., 2020; Berardi et al., 2021). BMD, BV/TV, BS/BV, Tb. N and Tb. SP are important indicators for evaluating bone function. To further demonstrate the protective effect of BH against RA, we used Micro CT as a supplementary test. Micro-CT imaging can accurately display lesion location and indicate the functional status of bone (Deng et al., 2021).

**FIGURE 6:**
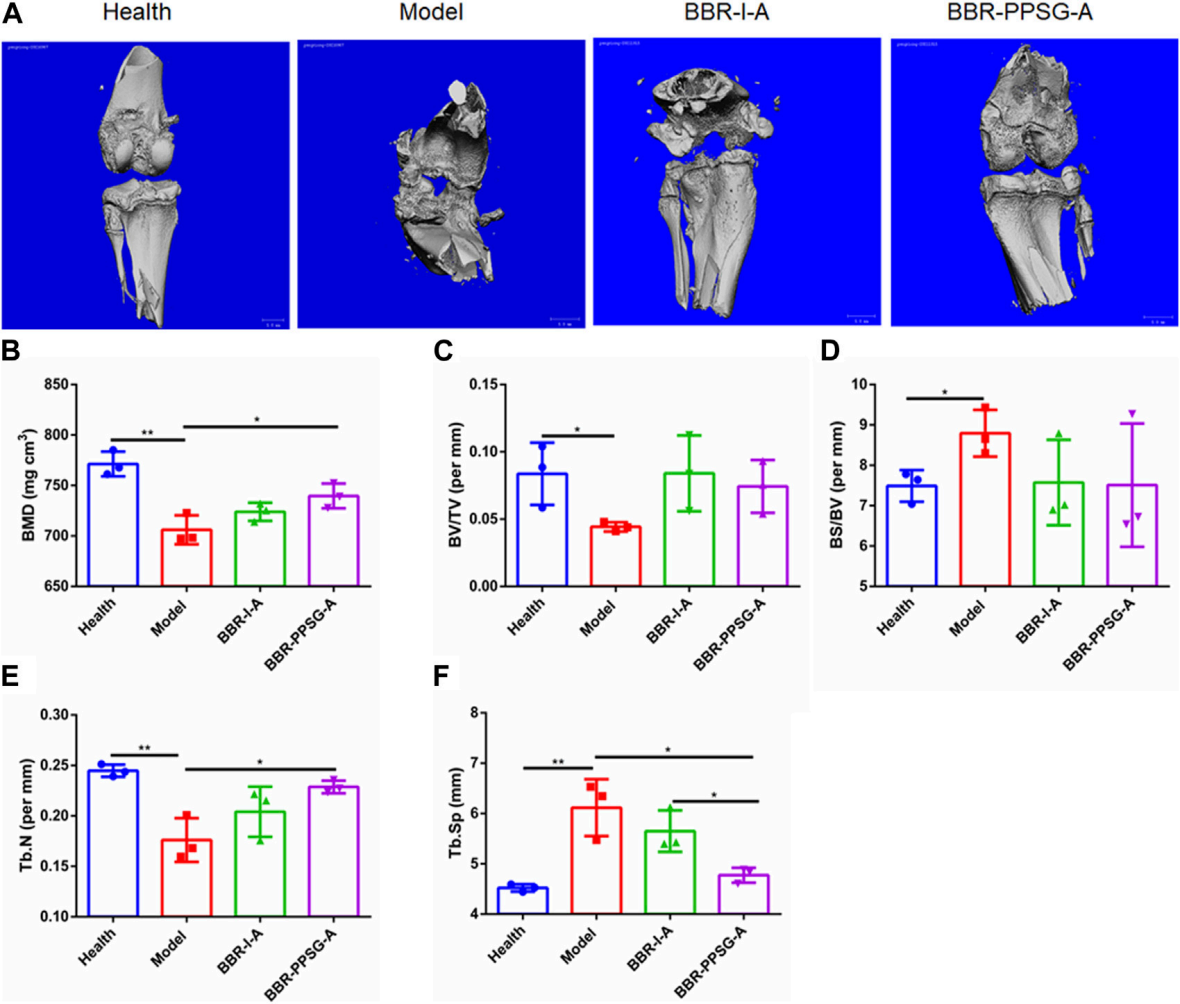
Bone function protection. **(A)** Micro-CT images of knee joints. **(B–F)** Quantitative micro-CT analysis of BMD, BV/TV, BS/BV, Tb. N, and Tb. Sp of the joints at the endpoint of the experiment. Data represent mean ± SD (n = 3).

OIA rabbits showed significant bone erosion and significant decrease in bone density (Figure 6A). Micro CT analysis showed that knee inflammation in the model group on day 30 was characterized by rough bone surface and severe bone erosion. Bone tissue image reconstruction in the BBR-I-A and BBR-PPSG-A groups was superior to that in the model group, and the surface was smooth, indicating that the degree of bone erosion was reduced to a certain extent (Figure 6A). Among all the parameters of bone function, there were statistical differences between the model group and the healthy group, indicating the success of modeling. In terms of efficacy, the data in each group of BBR-PPSG-A were close to the healthy group, and were improved compared with the model group. The three groups had statistical differences, indicating that BBR-PPSG-A can alleviate the loss of bone function in late-stage inflammation. Compared with BBR-I-A, four parameters of BBR-PPSG-A were better than those of BBR-I-A, with a statistically significant difference in 1 group. Combined with the above-mentioned cytokine detection results, compared with continuous injection of berberine solution for 28 days, the gel group injected only once has a better overall effect in controlling rheumatoid joints and achieves long-term sustained release effect.

## 4 Discussion

Berberine possesses various pharmacological effects; however, it has limitations in terms of solubility, absorption and biological distribution. Studies have shown that the low bioavailability of BBR is mainly attributed to two factors: poor water solubility, which hinders gastrointestinal absorption, and extensive (Liu et al., 2010). Many studies have focused on improving the bioavailability of BBR by improving formulation or delivery systems. Some studies have prepared nano-scale BBR by reducing the size of BBR particles, thereby improving the solubility of BBR, enhancing the efficacy and reducing the adverse reactions (Wang et al., 2015; Xie et al., 2017; Sahibzada et al., 2018). Other studies have loaded BBR onto drug delivery systems. Yu loaded BBR onto polymer–lipid hybrid nanoparticles (PEG–lipid–PLGA NPs) to improve the efficiency of oral BBR administration. This compound enhanced the liposolubility of BBR, and increased its relative oral bioavailability by 343% compared to BBR alone (Yu et al., 2017). Zhang et al. (2014) prepared berberine-phospholipid complex using TPGS 1000 and SiO2 as carriers. The berberine-phospholipid complex exhibited higher solubility and dissolution rate. Significant improvements in C_max_ and AUC_0→t_ were obtained and the relative oral bioavailability was increased by 322.66% compared to BBR. Additionally, some studies have prepared berberine as liposomes to enhance absorption. Lin developed polyethylene glycol (PEG) berberine liposomes, which effectively reduced the clearance rate of berberine in both plasma and tissues (Lin et al., 2013). Nguyen et al. (2014) prepared chitosan-coated BBR nano-liposomes, which exhibited better stability and slower BBR release kinetics. The aforementioned studies have all demonstrated effective enhancement of bioavailability by reducing the size of BBR particles, delivering BBR with carriers, or preparing BBR liposomes. The BBR-loaded phospholipid-based phase separation gel developed in this study not only increased the AUC and prolonged the half-life, but also exhibited sustained release properties.

An appropriate animal models is the basis for research on rheumatoid arthritis, which is a serious autoimmune disease that mainly leads to the destruction and inflammation of local joint tissues (Allen et al., 2018; Schneider and Krüger, 2013). Animal models of RA are usually induced by genetics, immunity, and environmental factors. In the process of induction, common methods include injecting substances such as anti-collagen, anti-DNA antibodies, lipopolysaccharides, bacteria and viruses to trigger abnormal response in the animal immune system, which could mimic the clinical symptoms and histopathology changes of human rheumatoid arthritis (Zhao et al., 2022). In animal research, mice, rats, rabbits, sheep, and other animals are often used (Yamashita et al., 2002; Abdalmula et al., 2014) In this study, rat and rabbit RA models were established respectively, in order to provide a more accurate theoretical and experimental basis for the prevention and treatment of rheumatoid arthritis.

The AIA rat model is widely used in the evaluation of antirheumatic drugs and rheumatoid arthritis, and its clinical and histological characteristics are similar to those of human rheumatoid arthritis, making it one of the most common animal models (Bevaart, et al., 2010). Therefore, the AIA rat model was first established in this study. Compared with healthy rats, the rats in the model group showed joint swelling and increased serum inflammatory cytokines IL-1β and TNF-α, which indicated that the AIA rat model was successfully established. Although the success rate of AIA model building is high, the maintenance time is relatively short (Huang S. M. et al., 2021; Zhao et al., 2022). Moreover, considering the differences in pathophysiology among various animal models of Freund’s adjuvant, including different morbidity, onset time and symptoms of arthritis, the rabbit RA model was included in our study, which was induced by OVA referring to Dumonde in 1962 (Dumonde and Glynn, 1962). Multiple subcutaneous injections of exogenous antigens promote the production of a large number of antibodies in the body, so that the antigens and antibodies are deposited in the synovium of the joint, activate the immune cells and complement system of the body, and eventually produce the antigen-antibody-complement immune complex, thus inducing local joint inflammation and joint swelling (Nandakumar, 2010).

This study first investigated the therapeutic effect of BBR-PPSG on RA rats. After confirming its favorable therapeutic effect on rheumatoid arthritis, BBR-PPSG was injected into the lesion site of RA rabbits to visually investigate the pathological and physiological changes of the lesion site. The pharmacokinetic and pharmacodynamic results of the two animal models both showed that BBR-PPSG could significantly reduce the level of inflammatory factors and improve the joint pathological status, and the curative effect lasted for about 1 month. Among RA rats, the results of the BBR-PPSG group were the closest to the health group, and both were better than the oral and injection groups. Compared with daily medication, BBR-PPSG not only significantly reduces the frequency of administration, but also ensures the therapeutic effect.

Cartilage and bone destruction are the main clinical features of RA, and protecting bone and cartilage from erosion is currently the main goal of treating rheumatoid arthritis. In the rabbit model, despite directly observing the therapeutic effect through joint cavity injection, the bone protection function of the preparation was also verified through CT imaging and CT data. The results of cytokines, histopathology and CT further confirmed the therapeutic effect of BBR-PPSG on RA. In the CT images, it is obvious that the joint bone morphology of the BBR-PPSG group was better than that of the injection group, which was greatly improved compared to the model group and was also reflected in specific bone functional parameters.

Except confirmed therapeutic effect, the advantages of this product preparation should be also highlighted. Phospholipid based phase separation gel, a solvent-induced *in situ* formation gel, is a novel sustained drug delivery system developed by our team in recent years (Zhang T. et al., 2015). The gel is simple, safe and consists of only phospholipid, ethanol, and injection grade oil. Convenient preparation with a lower cost, makes it suitable for industrial production preparation. Previous studies showed that PPSG has no significant cytotoxicity when co-cultured with L929 and HUVEC cell lines. Compared the PPSG content of each component, and ethanol at 70:15:15 has the slowest and least release rate, which contributes to control sudden release and effectively reduce adverse stimuli at the injection site. Besides, this proportion has high biocompatibility and can be biodegradable (Zhang X. et al., 2015; Zhang et al., 2019a). Therefore, we did not conduct the study of each proportion, and directly used the optimal proportion.

The sol-gel transformation process of PPSG is from outside to inside, which is completed in 12 min in 37°C PBS buffer. About 4 h after subcutaneous injection in rats, the gel turns to a coagulated state completely (Zhang et al., 2019b). Under atomic microscopy, the liquid PPSG was uniform, indicating that the phospholipids in the PPSG were completely dissolved. Images of solidified PPS clearly displayed phospholipid precipitate particles, which can act as repositories for the sustained release of drugs after phase transformation when PPSGS exposed to water (Zhang et al., 2019b). Compared with berberine solution by oral or subcutaneous injection, the gel shows a slower release rate *in vivo*, which can prolong action time and reduce toxicity. Moreover, BBR-PPSG also provides good stability and can be stored for a long time within a certain temperature range.

## 5 Conclusion

In this study, we developed and validated a BBR-PPSG system for the treatment of RA. A single dose of BCR-PPSG could be released smoothly and continuously in animals for 1 month. By establishing adjuvant induced arthritis model in rats and ovalbumin induced arthritis model in rabbits, we showed BBR-PPSG could effectively relieve joint swelling, inflammatory cell infiltration, reduce the levels of TNF-α and IL-1β, and maintain the stability of synovial chondrocytes. Furthermore, Micro-CT analysis of bone erosion in OIA rabbits with inflammatory joints reveals that bone functional parameters in BBR-PPSG group were repaired and reversed. These results suggest that BBR-PPSG has the potential for long-term inflammatory relief and bone recovery against RA.

## Data Availability

The original contributions presented in the study are included in the article/supplementary material, further inquiries can be directed to the corresponding author.
